# Appeal of the Philadelphia Obstetrical Society

**Published:** 1873-05-01

**Authors:** 


### APPEAL OF THE PHILADELPHIA OBSTETRICAL SOCIETY

FOR AID IN THE FORMATION OF A MUSEUM OF DISTORTED PELVES, OBSTETRICAL AND GYNŒCOLOGICAL INSTRUMENTS.

At a recent meeting of the Philadelphia Obstetrical Society, it was decided to establish a museum, for the collection of deformed and distorted pelves, and for the preservation of obstetrical instruments possessing historical value, or illustrating new methods of treatment.

The Society was led to take this action for three reasons :

1. No subject at present possesses more interest to the obstetrician than the study of the various deformities of the pelvis, their mode of production, their influence on the process of parturition, and the principles which should guide the accoucheur when operative interference is deemed necessary. The profession is gradually becoming more and more convinced of the influence of contractions, more or less marked, in causing not only tedious and difficult labors, but also in the production of the mal-presentations and of many of the accidents of labor.

At present there does not exist any extensive collection of female pelves, by which a comprehensive study of this subject can be successfully undertaken. Feeling, therefore, the importance of such observation, the Society proposes to establish a museum having this object in view, and would therefore earnestly solicit such specimens of contracted pelves as may be in the possession of members of the medical profession, who may be willing to yield the pleasure of individual possession in order to assist in forming a collection which will allow a wider and more comprehensive survey of this subject. If the original specimen cannot be sent, casts or photographs are solicited. In certain cases possessing unusual interest, the Society is prepared to offer a pecuniary recompense, should this be desired.

2. Recognizing the fact that various instruments designed for obstetric manipulations, or for the performance of operations in uterine surgery, having been superseded by new and improved models, now possess only an historical interest, the Society has determined to collect such instruments and preserve them, as illustrating the progress of this branch of our art in America. We would also warmly urge upon the inventors of new or modified instruments, and upon surgical instrument makers in general, the desirability of presenting to the Society specimens of intruments, pessaries, and special mechanical contrivances, which they may be desirous of bringing before the profession.

All objects, whether embraced in the first or second class, will be conspicuously placed in the museum of the Society, after having been labeled with an explanatory description, and with the name of the donor.

They will also be carefully preserved, and open to the inspection of all interested in the support and advancement of obstetrics and the kindred branches of medicine.

All objects for the museum may be sent to the Secretary of the society, Dr. J. V. Ingham, No. 1,342 Spruce street, Philadelphia, who will at once acknowledge their receipt, and will gladly furnish such additional information as may be desired.

By order of the Society,

WM. GOODELL, M.D., *Pres't.*

WM. F. JENKS, M.D.

J. V. INGHAM, M.D. } *Committee.*

HORACE WILLIAMS, M.D.

